# Evaluation of foliar application of selenium and flowering stages on selected properties of Iranian Borage as a medicinal plant

**DOI:** 10.1038/s41598-022-16241-z

**Published:** 2022-07-22

**Authors:** Mazaher Hosseinzadeh Rostam Kalaei, Vahid Abdossi, Elham Danaee

**Affiliations:** 1 Department of Horticultural Sciences, Aliabad Katoul Branch, Islamic Azad University, Aliabad Katoul, Iran; 2 Department of Horticulture and Agronomy Science, Science and Research Branch, Islamic Azad University, Tehran, Iran; 3 Department of Horticultural Sciences, Garmsar Branch, Islamic Azad University, Garmsar, Iran

**Keywords:** Plant physiology, Secondary metabolism

## Abstract

Many of the active constituents of drug or medicines were originally derived from medicinal plants. Iranian Borage are still being used in regular basis. Selenium (Se) is an essential mineral nutrient for animal and human growth. The aim of this research was to determine the effect of (2, 4, 8 and 16 mg L^−1^) of as sodium selenate (Na_2_SeO_4_) and as sodium selenite (Na_2_SeO_3_) on some important properties of Iranian Borage in factorial based on Randomized Complete Block Design via four steps: 2 true leaves stage, ten leaves, 2 weeks and 1 week before flowering. The traits were evaluated during flowering period. Results showed that the highest shoot fresh and dry weight and shoot length, total alkaloid, essential oil percentage were obtained by 4 mg L^−1^ sodium selenate at the end of flowering. In addition, 4 mg L^−1^ sodium selenate concentration significantly improved flower yield (diameter, number, weight). The plants were treated with 8 mg L^−1^ sodium selenate, the higher total phenols and flavonoids, antioxidant activity, soluble sugars, root and fresh weight was seen at end of flowering. When the plants were sprayed with 4 mg L^−1^ sodium selenite higher total chlorophyll was observed at full of flowering. 16 mg L^−1^ sodium selenite released the maximum Se acclimation in the petals. 20 composites were discovered containing ɑ-Pinene (23.61%) with sodium selenate in 4 mg L^−1^. Generally, selenium sources significantly improved morpho-physiological and phytochemical.

## Introduction

Nontoxic heavy metal concentrations in medicinal and aromatic plants (MAPs), which have significant contents of some functional microelements, such as zinc (Zn), selenium (Se), and iron (Fe), are desired for daily diets^1^. Selenium is essential element for human and animal health due to the activation of antioxidant defense systems^2^. In addition, it shows a narrow range between deficiency (< 40 μg day^−1^) and toxicity (> 400 μg day^−1^) in human nutrition^3^. Due to its ability to oxidize thiol groups in the virus protein disulfide isomerase, selenite may even prevent COVID-19 contagion^4^. The plants play an exclusive role in recirculation and releasing the Se from the soil to the food chain. Selenium is not considered an essential element in plants, but it elicits the production of secondary metabolites and increases the enzymatic and non-enzymatic antioxidant capacity. Thus, in addition to biofortification, Se enhances the levels of specific bio-active/health promoting compounds that may also have positive effects on the plant physiology and metabolism^5^. The Se concentration in agricultural products is dependent on the content in the soil and biological accessibility^6^. The Se observation in plants is dependent on the chemical forms, concentration and factors such as pH, salinity, calcium carbonate content and plants capability^7,8^. Selenite and selenate are the main forms of Se fertilizer. Selenate is more effective than selenite for Se application to soil for the purpose of biofortification, although it is more easily leached to deep soil by the water from irrigation and rainfall due to its higher mobility. Furthermore, in edible parts of plants, Se in organic forms is more effective to human and animals than that in inorganic forms. Kápolna et al.^9^ also showed foliar application of selenite enhanced bio-synthesis of organic Se species compared to selenate. Se increases plant tolerance to salinity^10^, drought^11^, cold^12^ and heavy metal^13^ stresses. The previous studies reported the beneficial effects of Se on seed germination^14^, enhancing crop yield^15^ and increasing the antioxidative capacity^16^ of the plants. The excitatory effects of the foliar application of Se on the growth characteristics of potato (*Solanum tuberosum*), roselle (*Hibiscus sabdariffa*), rye (*Secale cereale*) and lettuce (*Lactuca sativa*) have been reported^17–20^. A study by Malik et al.^21^ indicated that the aerial parts of mung bean (*Vigna radiata*) compared to the roots was more influenced by the sodium selenate application^13^. Foliar Se application is another commonly used technique for increasing the Se content in edible parts. Foliar Se application of selenite or selenate solutions significantly promoted the Se content in carrot roots and leaves, bulbs, leaves as well as radish flowers and leaves^22^.

Fruits, vegetables, and herbs are an important source of exogenous antioxidants, including polyphenols and selenium. The use of herbal products results in positive effects on the treatment of diseases caused by free radicals. *Echiuma moenum* Fisch. & Mey. (Iranian Borage) is a perennial plant from Boraginaceae family which dispersion is on Canary Island, Europe, West Asia, and North and South Africa. This plant is also dispersed in Iran and Caucasus^23^. *E. amoenum* in includes secondary metabolites such as anthocyanins, tannins, mucilage, flavonoids and alkaloids^24^. This medicinal plant has long been used as a tonic, tranquillizer, diaphoretic, a remedy for cough, sore throat and pneumonia in traditional medicine of Iran^25^.

Due to the Se deficient areas, this issue has been considered in some developed countries and achieved some success by introducing selenium into their native plants, but in the mentioned country this problem has been less addressed and almost less action has been taken in this regard. Since different physiological traits related to Se have not been discussed in detail on Iranian Borage as a medicinal plant, the aim of this study was to investigate the effect of different stages of flowering and sodium selenate and sodium selenite applications on some morpho-physiological and phytochemical properties of the *E. amoenum*.

## Materials and methods

### Plant material

**Figure 1:**
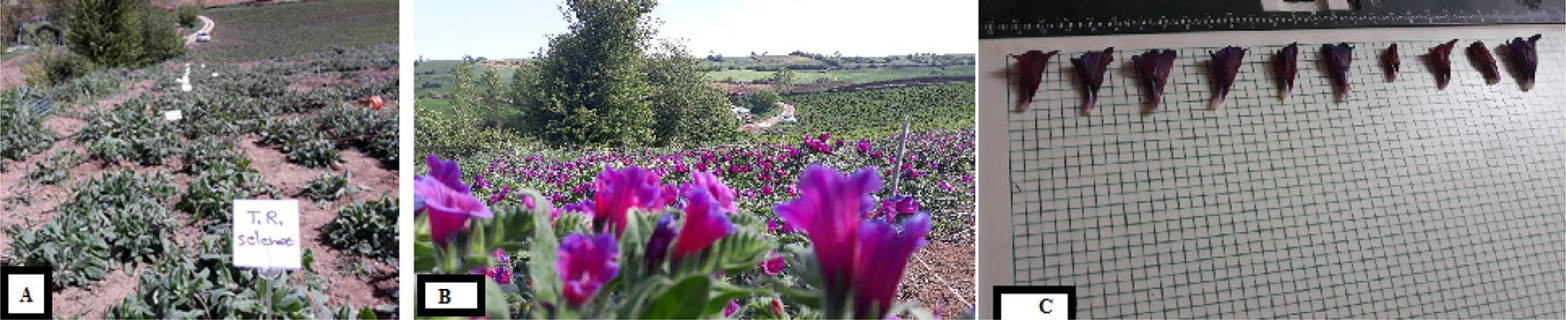
*Echium amoenum* treatead with Selenate (before flowering) (**A**); (after flowering) (**B**); Sample of flower treated with different selenium sources (**C**).

The experiment was conducted as a factorial based on Randomized Complete Block Design (RCBD) with 3 replications in Parm village, Nemarstagh section, Behshahr city, Mazandaran, Iran (36° 41′ 32.46″ N, 53° 33′ 9.43″ E). The Se solution was sprayed as sodium selenate or sodium selenite solution (Sigma Aldrich, colorless crystalline powder) at concentrations of 2, 4, 8 and 16 mg L^−1^ in four steps in the form of foliar application: 2 true leaves stage, ten leaves, 2 and 1 weeks before flowering of *E. amoenum*. Each plant was treated with 250 mL of Se-enriched solution, whereas each control plant was sprayed with 250 mL of distilled water^26^ (Fig. 1A,B).

### Determination of growth parameters

In this study, several growth parameters of plants were evaluated, including flower number, length and diameter of flowers (with collis) and fresh and dry weight of flowers/areal parts/roots (digital scale). Shoot length was recorded with ruler (Fig. 1C).

### Determination of physiological and phytochemical parameters

Furthermore, several physiological and phytochemical parameters were measured, including chlorophyll and carotenoid content, antioxidant activity, total phenols and flavonoids contents, soluble sugars content, petal anthocyanin, total alkaloid contents in leaves and essential oils and Se content in petals.

#### Chlorophyll contents

100 mg of flower tissue in fractions were placed in a vial containing 7 mL (Dimethylsulfoxide) DMSO, and chlorophyll was extracted into the fluid without grinding at 60 °C by incubating for 25 min. The extract was transferred to a new tube and was increased to a total volume of 10 mL with DMSO. Measurements of the chlorophyll and carotenoid contents were performed using the spectrophotometric method at a wavelength of 645, 663 and 470 nm against a blank DMSO (Jenway 7305 UV–vis spectrophotometer, England)^27^.

#### Antioxidant activity, total phenols and flavonoids contents

##### Extraction

30 g of the plant powder were weighed separately and accurately and then extracted in a Soxhlet Apparatus using thimble in order to get the best extract. Various solvents were used depending upon their polarity index with increasing polarity (DCM, Methanol and Water).

5 g of the sample was weighed into a 250 mL beaker and 200 mL of 10% acetic acid in ethanol was added and covered and allowed to stand for 4 h. This was filtered and the extract was concentrated on a water bath to one-quarter of the original volume. Concentrated ammonium hydroxide was added dropwise to the extract until the precipitation was complete. The whole solution was allowed to settle and the precipitated was collected and washed with dilute ammonium hydroxide and then filtered. The residue is the alkaloid, which was dried and weighed.

##### Antioxidant activity

Determination of antioxidant activity was performed by using the DPPH (2,2-diphenylpicrylhydrazyl, Sigma, Aldrich) test. Samples’ absorptions were read at a wavelength of 517 nm with spectrophotometer^28^.

##### Phenols content

Measuring the total phenols was performed according to the Folin’s reagent method and the use of gallic acid as standard by using a spectrophotometer at the wavelength of 765 nm^28^. The method proposed by Menichini et al.^29^ was used for measuring the total flavonoid by means of a spectrophotometer at a wavelength of 510 nm through a standard curve of quercetin.

##### Soluble sugars content

The content of soluble sugars in the samples was determined as followed, 100 mg of the samples were completely powdered with liquid nitrogen and 10 mL of 80% ethanol was added and transferred into a centrifuge tube. The tubes were centrifuged at 5000 rpm for 10 min. The supernatant was then poured into Erlenmeyer with 10 mL of 80% ethanol. It was centrifuged again at 5000 rpm for 10 min and the supernatant was added to the previous Erlenmeyer flask. In the next step, 25 µL of the desired solution was poured into a plate, after which 25 µL of 5% phenol solution was added to the samples. Then 125 μL of concentrated sulfuric acid was transferred to them. Finally, the soluble sugars content was read at a wavelength of 490 nm using a spectrophotometer (Jenway 7305 UV–Vis spectrophotometer, England) according to the phenol–sulfuric acid method, while glucose (Sigma-Aldrich, USA) was used as standard^30^.

##### Total alkaloid contents in petals

Total alkaloid content (TAC) in the extract was evaluated using standard method. Flower extraction (2.50 g) were taken in a beaker in which 200 mL of 10% CH3COOH in methanol was added and were incubated for 4 h at RT. Then, concentrated NH_4_OH was added dropwise until complete precipitation. Subsequently, supernatant was removed and precipitates were washed with 20 mL of 0.1 M of NH_4_OH. Lastly, Total alkaloid contents in petals was read at a wavelength of 258 nm using a spectrophotometer, while atropine (Fluka Art. 11320, Merck, Germany) was used as standard^31^.

##### Selenium content in petals

For measurement of Se content in petals, initially 1 g from dried petals samples were digested in 5 mL mixture of nitric acid and concentrated perchloric acid (with volume rate of 4:1) at temperature 130 °C for an hour. After cooling, 5 mL of concentrated hydrochloric acid were added and heated for 20 min at a temperature of 115 °C. Finally, atomic spectrometry (ICP-OES spectrometer Integra XL2, GBC Australia) was used to determine of selenium contents in petals^32^.

##### Essential oils

In order to measure the EO content, 100 g of dried aerial parts from each treatment were hydrodistilled in the Clevenger type apparatus for 3 h, and reported as v/w percentage (European Pharmacopoeia 1983). The EO yield was measured with multiplying the EO content with the plant yield of the experimental treatments. To identify the essential oil of this plant, the mass spectrometer attached to the chromatograph gas was used^33^.

### Statistical analysis

Data are presented as mean values ± standard deviation (S.D.) at 3 replications. Data were analyzed by using Duncan’s multiple range test (*p* < 0.05) by SAS, version 9.4.

### Ethical statement

“This research received no specific grant from any funding agency in the public, commercial, or not-for-profit sectors.” “The authors declare that there is no conflict of interest regarding the publication of this article.” “All procedures followed were in accordance with the ethical standards of the responsible committee on human experimentation (institutional and national). Informed consent was obtained from all patients for being included in the study.” All authors contributed to the study conception and design. Material preparation, data collection and analysis were performed by [Mazaher Hosseinzadeh Rostam Kalaei], [Vahid Abdossi] and [Elham Danaee]. The first draft of the manuscript was written by [Vahid Abdossi] and all authors commented on previous versions of the manuscript. All authors read and approved the final manuscript. Vahid Abdossi and Elham Danaee; Conceptualization, Methodology, Formal analysis and investigation, Vahid Abdossi; Writing-original draft preparation, Mazaher Hosseinzadeh Rostam Kalaei, Vahid Abdossi and Elham Danaee; Writing—review and editing, Vahid Abdossi and Elham Danaee; Resources, Supervision.

## Results

### The effect of flowering stages and Se forms on morphological properties

The results showed that the plants had the highest number of flowers in the full flowering stage and the lowest number of flowers in the end of flowering stage. Foliar application of 4 mg L^−1^ of sodium selenate compared to the other concentrations significantly increased the flower number. As the highest amount of flower number with an average 290.33 was observed after the application of such treatment and in the full flowering stage (Table 1).

**Table 1:** The effect of selenium sources and flowering stages on some morphological characteristics of *Echium amoenum.*

Flowering time × (treatment) Se (mg L^−1^)		Shoot length (cm)	Shoot fresh weight (g)	Shoot dry weight (g)	Root fresh weight (g)	Root dry weight (g)	Flower number	Petal alkaloid (mg/100 g DW)	Flower fresh weight (g)	Flower dry weight (g)
B.F	0 Con	37^mn^	200^o^	39^mn^	151^mn^	14^mn^	106^no^	3.13^eh^	11^gh^	3.06^gh^
B.F	2 Ssit	41^lm^	217l^n^	42^lm^	159^lm^	15^lm^	119^m^	3.28^cg^	18^cf^	4.7^def^
B.F	4 Ssit	45.66^k^	236^lm^	45^kl^	161^klm^	15.3^klm^	121^lm^	3.66^bc^	20^cde^	5.4^cde^
B.F	8 Ssit	38^mn^	227^mn^	44^kl^	175^j^	16^j^	111^n^	3.5^be^	18^cf^	4.81^cf^
B.F	16 Ssit	30^o^	194^o^	37^n^	143^n^	13^n^	103^o^	3.14^dh^	9^h^	2.46^h^
B.F	2 Ssat	43.3^kl^	245^l^	47^k^	166^jkl^	15^jkl^	111^n^	3.84^b^	15^efg^	4.09^efg^
B.F	4 Ssat	58^j^	269^k^	52^j^	174^j^	16 ^j^	126^l^	4.23^a^	16^efg^	4.21^efg^
B.F	8 Ssat	43^kl^	248^l^	48^k^	198^hi^	19^hi^	109^no^	3.57^bcd^	17^def^	4.4^efg^
B.F	16 Ssat	34^n^	196^o^	38^n^	147^n^	14^n^	104^o^	3.36^cf^	10^h^	2.6^g^
F.F	0 Con	64.66^ghi^	529^j^	103^ghi^	175^j^	16.6^j^	176^fg^	2.4^k^	21^cde^	5.4^cde^
F.F	2 Ssit	69.16^fgh^	542^i^	105^fgh^	191^i^	18^j^	189^e^	2.83^gk^	31^ab^	8.11^ab^
F.F	4 Ssit	64^ef^	569^fg^	109^fg^	190^i^	18^i^	204^d^	3.08^ei^	32.7^ab^	8.4^ab^
F.F	8 Ssit	64^ghi^	559^gh^	108^ef^	204^h^	19^h^	180^f^	2.61^jk^	30^b^	7.7^b^
F.F	16 Ssit	63.2^de^	523^jk^	100^i^	170^jk^	16^jk^	168^hi^	2.73^hk^	17^def^	4.5^def^
F.F	2 Ssat	70.66^hi^	553^hi^	108^ef^	201^h^	19^hi^	227^c^	2.86^gj^	23^c^	6.14^c^
F.F	4 Ssat	77.33^ed^	576^g^	115^d^	196^hi^	18.6^hi^	290^a^	2.5^jk^	35^a^	9.18^afgh^
F.F	8Ssat	74^ab^	558^gh^	107^efg^	263^g^	25^g^	245^b^	2.39^k^	30^ab^	7.9^ab^
F.F	16 Ssat	61^bcd^	528^j^	102^hi^	171^j^	16^i^	161^c^	2.82^gk^	18^cf^	4.84^cf^
E.F	0 Ssit	63.33^ij^	619^cde^	120^c^	590^f^	56^f^	150^j^	2.66^ik^	17^def^	4.5^def^
E.F	2 Ssit	68.33^efg^	628^cd^	121^c^	611^e^	58^e^	160^k^	2.9^gj^	18^cf^	4.74^cf^
E.F	4 Ssit	72.33^dcde^	631^c^	123^c^	625^d^	60^d^	170^j^	2.91^gj^	21^cde^	5.44^cde^
E.F	8 Ssit	65.66^fgh^	630^c^	121^c^	667^c^	63^c^	162^gh^	2.77^hk^	20^cde^	5.4^cde^
E.F	16 Ssit	63.7^hi^	608^e^	119^c^	585^f^	55^f^	144^ij^	2.93^fj^	16^efg^	4.2^efg^
E.F	2 Ssat	70.66^ed^	673^b^	130^b^	675^c^	64^c^	181^f^	2.71^hk^	19^cf^	5.11^cf^
E.F	4 Ssat	80.66^a^	701^a^	137^a^	705^b^	67^b^	241^b^	3.1^ei^	22^cd^	5.88^cd^
E.F	8 Ssat	76.33^bc^	678^b^	131^b^	773^a^	73^a^	198^d^	2.82^hk^	18^cf^	4.85^cf^
E.F	16 Ssat	63^hi^	615^de^	119^c^	585^f^	55^f^	146^k^	2.77^hk^	14^fgh^	3.72^fgh^

Flowering time × (treatment) Se (mg L^−1^)		Se accumulation (petal) (g/kg DW)	Phenol (mg GAE/g DW)	Flavonoid (mg/QE/g DW)	Antioxidant activity (%)	Total sugar (mg/g DW)				
B.F	0 Control	0.045^mn^	10.53^mn^	2.36^k^	15^kl^	1.71^fg^				
B.F	2 Ssit	0.049^lm^	10.17^mn^	2.35^k^	15^kl^	2.07^efg^				
B.F	4 Ssit	0.056^kl^	11.02^lm^	3.05^k^	15^kl^	3.73^cd^				
B.F	8 Ssit	0.072^eh^	10.33^mn^	2.36^k^	15^kl^	2.42^efg^				
B.F	16 Ssit	0.09^c^	9.33^n^	2.1^6k^	15^kl^	1.5^g^				
B.F	2 Ssat	0.09^c^	13.66^k^	6.21^j^	16^k^	3.69^cd^				
B.F	4 Ssat	0.096^c^	12.09^l^	6.57^ij^	21^hi^	3.92^c^				
B.F	8 Ssat	0.114^b^	23^c^	15.04^c^	36^c^	7.38^a^				
B.F	16 Ssat	0.124^a^	9.33^n^	2.23^k^	13^l^	1.6^fg^				
F.F	0 Con	0.034^k^	15.4^ij^	6.7^ij^	20^hij^	1.95^fg^				
F.F	2 Ssit	0.039^nop^	15.1^1j^	5.34^j^	20^hij^	2.29^efg^				
F.F	4 Ssit	0.042^mno^	16^hij^	6.03^j^	21^hi^	4.21^c^				
F.F	8 Ssit	0.066^fi^	15.11^ij^	7.64^j^	21^ghi^	3.03^de^				
F.F	16 Ssit	0.07^ef^	12^l^	5.94^hi^	20^ij^	1.66^fg^				
F.F	2 Ssat	0.06^gi^	16^ghi^	9.2^j^	23^fgh^	3.91^cd^				
F.F	4 Ssat	0.072^fi^	11^gh^	11.66^g^	35^c^	4.06^c^				
F.F	8Ssat	0.078^ef^	27^b^	18^b^	43^b^	7.6^a^				
F.F	16 Ssat	0.081^de^	12.33^l^	6^j^	18^jk^	1.93^fg^				
E.F	0 Ssit	0.023^d^	20^ef^	10.56^ef^	27^de^	2.3^efg^				
E.F	2 Ssit	0.033^q^	20^f^	8.32^gh^	26^def^	2.51^ef^				
E.F	4 Ssit	0.036^p^	21^def^	9.02^gh^	27^d^	4.4^c^				
E.F	8 Ssit	0.056^op^	20^f^	10.69^ef^	26^def^	4.46^c^				
E.F	16 Ssit	0.07^jkl^	17^gh^	8.92^gh^	24^efg^	2.27^efg^				
E.F	2 Ssat	0.06^fi^	21^c^	12.18^f^	37^c^	4.12^c^				
E.F	4 Ssat	0.064^hij^	22^cde^	17.66^b^	37^c^	5.45^b^				
E.F	8 Ssat	0.065^fi^	32.38^a^	21^a^	52^a^	7.82^a^				
E.F	16 Ssat	0.072^efg^	17^g^	9.66^fg^	25^def^	2.22^efg^				

^†^Means within each column followed by the same letter are not different according to the Duncan test.

*B.F* Beginning of flowering, *F.F* Full flowering, *E.F* End of flowering, *Ssit* sodium selenite, *Ssat* sodium selenate.

In this experiment, flower diameter was affected by flowering stages and Se forms. Average flower diameter at the beginning, full flowering and end of flowering was 1.59, 1.92 and 1.78 cm, respectively. The highest flower diameter was observed 2.13 and 2.02 respectively after treatment 4 mg L^−1^ of sodium selenate and sodium selenite (Table 1).

In this research, the fresh and dry weights of flowers were significantly affected by flowering stages and selenium forms. The results showed that the yield of fresh and dry weight of flower has the lowest amount at the beginning of flowering and highest amount at the full flowering stage. Foliar application of 4 and 8 mg L^−1^ sodium selenate or 2 and 4 mg L^−1^ sodium selenite at the full flowering stage significantly increased the fresh and dry weights of flowers compared to other treatments (Table 1).

In the present research, the fresh and dry weights of shoots was affected by the flowering stages. The maximum shoot fresh weight was 701 g, 6.13 with 4 mg L^−1^ selenate and 673 and 678 at 2, 8 mg L^−1^ selanate, (678 g, 673 g) respectively. In addition, the form and concentration of Se had significant effect on shoot dry weight of *E. amoenum*. Its highest amount with an average of 137 g was observed after 4 mg L^−1^ sodium selenite treatment which showed a significant difference with other experimental treatments (Table 1).

The application of sodium selenate and sodium selenate treatments in the low concentrations significantly increased the shoot length compared to the control treatment. As the application of 4 mg L^−1^ sodium selenate increased the shoot length up to 80.66 cm. The results indicated that the application of the higher concentrations of sodium selenate and sodium selenite (16 mg L^−1^) significantly decreased the shoot length compared to the control treatment (Table 1).

In this study, foliar application 8 mg L^−1^ sodium selenate significantly increased the amount of fresh and dry weights of roots. This treatment increased the amount of this traits 773 g at the end of flowering. In addition to that, the lowest amount was for the foliar application of the plants with 16 mg L^−1^ sodium selenate treatment. The results showed that the amount of root dry weight reach to its lowest at the beginning flowering stage and the contents there of are added by approaching to the end of flowering stage. The highest amount with the averages 73 g and 067 g was for the period of harvesting flower at the end of flowering and foliar application 8 and 4 mg L^−1^ sodium selenate, respectively (Table 1).

### The effect of flowering stages and Se forms on physiological and biochemical properties

**Figure 2:**
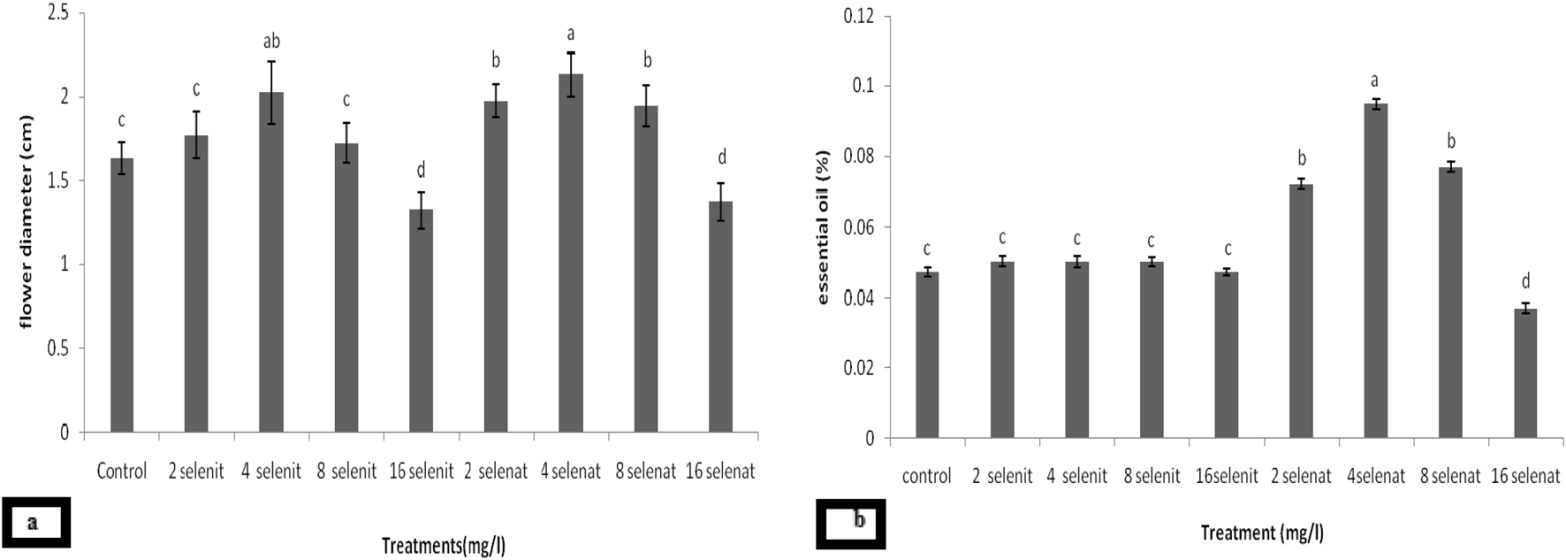
The effect of selenium sources in full flowering stages on flower diameter (cm) (**a**) and essential oil (%) (**b**) of *Echium amoenum*. Different capital letters at the same specie indicate differences between treatment according to the Duncanʼ test.

In this study the content of total chlorophyll was significantly affected by the flowering stages. The plants at the beginning of flowering (1.68 mg g^−1^ FW) and the end of flowering (1.68 mg g^−1^ FW) had higher content of the total chlorophyll. Moreover, the form and concentration of selenium had significant effect on the amount of total chlorophyll in *E. amoenum*. The highest amount of total chlorophyll was observed after 4 mg L^−1^ sodium selenite treatment and then 8 mg L^−1^ sodium selenite (Fig. 2a).

Antioxidant capacity in *E. amoenum* was significantly affected by the flowering stages and selenium forms. The results showed that the antioxidant activity gradually increased significantly from the beginning of flowering to the end of flowering. The highest amount of antioxidant activity in the plant with an average of 52% was observed at the end of flowering stage and after 8 mg L^−1^ sodium selenate application (Table 1).

The amount of total phenol significantly increased from beginning to the end of flowering. Foliar application of 8 mg L^−1^ of sodium selenate and harvesting at the end of flowering significantly increased the content of the total phenol (by 32.38 mg gallic acid/g DW) compared to other treatments (Table 1).

Total flavonoid contents had the lowest amount at the beginning of flowering and highest amount at the end of flowering. The results indicated that the plants which sprayed with 8 mg L^−1^ of sodium selenate and harvested at the end of flowering stage, had the highest amount of the total flavonoid (by 21.01 mg quercetin/g DW) (Table 1).

According to Table 1 the highest amount of soluble sugars was achieved after 8 mg L^−1^ sodium selenate application. There was no significant difference between the flowers harvested at the different stages of flowering after this treatment.

The results indicated that total alkaloid in petals at the beginning of the flowering was more than other stages. 4 mg L^−1^ sodium selenate foliar application significantly resulted in increasing the total alkaloid contents in leaves and petals. As after the application this treatment, the amount of the total alkaloid in leaf and petal reached to 54.33 and 4.53 mg/100 g DW (Table 1).

Generally, by increasing the growing period of the plants, the content of Se in the petals was reduced. The results showed that by increasing the concentration of the selenium sources used, the content of this element in petals was also increased significantly. Among the used sources, the effect of sodium selenate was more than sodium selenite. As the highest amount of Se in the petals 0.124 g kg^−1^ DW was achieved after the application of 16 mg L^−1^ sodium selenate treatment (Table 1).

At the present study, the total essential oil was affected by the flowering stages. The maximum average at the full of flowering was 0.076%. Moreover, the amount of total essential oil in flowers was changed after the application of forms and different Se concentrations. After the application of 4 mg L^−1^ sodium selenate, the highest amount was achieved by 0.1%. In addition, the lowest amount of essential oil (0.037%) was related to 16 mg L^−1^ sodium selenate treatment (Fig. 2b).

Using GC and GC–MS 33 constituents were discovered 20 composites in Table 2. Results demonstrated that Se increased some major oil components include δ-Cadinene and a-Pinene. The effectiveness of dill essential oil is indicated by the flowering time and Selenium forms. Our finding also illustrated that essential oil generate from plant under treatment with Selenium exhibited a dose dependent.

**Table 2:** The effect of selenium sources and flowering stages on some essential oils of *Echium amoenum.*

	Compsition	RI	Con	Sodium selenite (mg L^−1^)				Sodium selenite (mg L^−1^)			
				2	4	8	16	2	4	8	16
1	a-Pinene	**922**	**18.11**	**19**	**19.02**	**20.2**	**20.73**	**21.23**	**23.61**	**21.23**	**19.19**
2	Myrcene	989	0.51	0.61	0.35	0.55	0.42	0.49	0.35	0.47	0.61
3	(Z)-β-Ocimene	1034	1.21	1.23	1.11	0.9	0.87	1.25	0.5	1.02	1
4	1-Octen-3-yl acetate	1111	0.56	0.61	0.68	0.12	1.25	0.98	0.4	1.11	2.21
5	Camphor	1167	0.8	4	3.8	3.3	2.2	1.2	0.9	2.1	1.9
6	Terpinen-4-ol	1180	1.23	0	–	–	–	–	–	–	–
7	Borneol	1184	4	1.6	1.2	0.8	1.6	1.3	1.7	2.08	1.44
8	Carvacrol	1307	4.6	4.4	4.36	4.86	4.77	5	5.89	4.22	4.08
9	δ-Elemene	1340	2.33	2.25	2.12	2.09	2.42	2	0.7	2.29	2.72
10	β-Elemene	1392	3.61	–	–	–	–	–	–	–	–
11	β-Caryophyllene	1413	2.01	1.55	2.65	2.02	2.21	1.61	1.79	1.87	3.08
12	α-Guaiene	1439	0.61	0.31	0.53	1	0.41	0.46	0.22	0.25	0.52
13	β-Sesquiphellandrene	1512	–	3.31	3.23	3.69	3.45	3.61	1.89	3.56	3.33
14	δ-Cadinene	**1519**	**46.66**	**47.31**	**47.74**	**47.91**	**49**	**50.02**	**53**	**50.5**	**47**
15	α-Cadinene	1533	4	4.3	4.26	4.5	3.54	4.03	5.5	4.11	4.23
16	Spathulenol	1577	1.21	–	–	–	–	–	0.02	–	–
17	β-Eudesmol	1651	0.77	0.64	0.56	0.68	0.78	0.91	0.25	0.67	0.85
18	α-Cadinol	1566	1.21	1.32	1.5	1.24	1.23	1.1	1.03	1.11	2.25
19	β-Bisabolol	1688	3.12	2.22	2.08	2.51	2.42	2.55	1.2	1.1	2.22
20	2-Phenethyl benzoate	1798	3.11	2.25	2.26	2.11	2.31	2.13	1	1.45	2.09
All (%)			99.65	96.91	97.45	98.48	99.61	99.87	99.95	99.14	98.72

Significant values are in bold.

Sodium selenate in 4 mg L^−1^ concentration increased ɑ-Pinene (23.61%) significantly compared to control plants. Comparison of average showed that the application of above-mentioned treatment significantly enhanced the δ-Cadinene rate (53%), if that high (16 mg L^−1^ led to a reduction in this combination (47%). Sodium selenite level resulted in a ɑ-Pinene (20.73%) compared to the treatments. Mean comparison figure out that highest percentage of δ-Cadinene (49%) was realized in treatment with 16 mg L^−1^ Sodium selenite (Table 2).

## Discussion

This study was conducted to test the effects of Se addition on the selected traits of *E. amoenum*.

The results illustrated that when various rates of Se were applied, the Se added to the nutrient solution was changed chlorophyll contents. Previous study conducted by Saffar Yazdi et al.^34^ different Se concentrations improved the morphological characteristics and enhanced chlorophyll pigments in *Spinacia oleracea*. In another research, the application of Se at low amount on rice resulted in increasing the photosynthesis rate and chlorophyll which is in compliance with our results^35^. In addition, the using Se had significantly increased transpiration level, photosynthesis rate, and stomatal conductance in grain sorghum^36^. High concentrations of Se induced a strong reduction of light energy absorbed by the antenna system (ABS/CS), of the stream of excitation energy (TRo/CS) and a decrease of electron transport through PSII (ETo/CS) in all varieties of wheat^37^. In a research conducted on cucumber the Se application resulted in increasing the photosynthesis pigments which is in compliance with the results of this research^10^. Several studies have confirmed an increase in chlorophyll content in plants after Se application^21^. Because the iron element is involved in increasing chlorophyll biosynthesis, Se is likely to increase the content of this pigment in the plant by increasing iron absorption^38^.

With dual effects on plants, the proper rate of Se application stimulated plant growth, whereas excessive Se application inhabited plant growth. The increasing in biomass and growth observed in the plants suggests that this Se treatment could be used to enhance *E. amoenum* improvement. The effect of Se on plant growth depends on rate and crop variety. For example, the total dry matter of plants was higher in Se-sprayed plants than that in controls for cultivar Monivip^39^. The effects of the Se foliar application on improving the growing performance can be the result of increasing the starch accumulation in chloroplasts^15^ and protecting the cell content like pigments^18^. The increased photosynthetic products of the plants treated by the Se can be the consequence of increasing the number of photoreceptors and consequently more synthesis of carbohydrates for growing the organ acts as a source for Se and carbohydrates^20^. The Se can play a role on increasing the quantum efficiency of photosystem II in the plants^40^. Improving the yields of the potato plants treated by the Se has indicated that the Se has probably been a factor to allocate more photosynthetic material to the tubers^20^. Researchers have found that higher levels of Se can reduce the rate of photosynthesis by destroying chloroplasts and disrupting the function of sulfur amino acids such as cysteine and methionine, thereby increasing the accumulation of more starch in the plant^35^. In the same research, Xue et al.^18^ attributed the lettuce’s growing and photosynthesis indexes to the Se role in producing the carbohydrate combinations. Malik et al.^21^ stated that the activity of starch hydrolyzing enzymes was stimulated significantly with Se. It was concluded that increase in growth of shoots and roots by application of Se was possibly the result of carbohydrate metabolism thus providing energy substrates for enhanced growth. This result is not consistent with experiments which showed that Se addition to the nutrient solution did not affect the biomass production of maize seedlings^39^, and lettuce^41^. However, several studies showed that Se application positively affected the plant. In a pot experiment, the Se-treated potato plants produced higher yields than did the control plants, which was related to its antioxidative effect in delaying senescence^42^. Similarly, in a hydroponic experiment’s treatment was associated with a 43% increase in Brassica production which was attributed to higher total respiratory activity in leaves and flowers^43^.

The results of this research showed that the application of Se sources results in significant increasing of selenium content in petals. The content of selenium in plants depends not only on the abundance of this element in the ground, but largely on its bioavailability pronounced by its chemical form, pH and redox potential of soil, various stages of plant development, presence of organic substances, activity of soil microorganisms, and climatic factors as well^8^. Meanwhile, the role of sodium selenate was more considerable. The findings of Ameriyan et al.^44^ on the onion showed the amount of bulb total Se was enhanced by increasing the sodium selenite and sodium selenate concentrations in the nutrient solution. However, this increase was higher in plants treated with sodium selenate, which supports the results of this study. Moreover, by increasing the Se levels, Se concentration in the tissues was increased^45^.

There is limited information about the absorption mechanism of selenite in the plants. It is suggested that selenite absorption mechanism is not metabolically dependent by the plant roots. Although previous studies Li et al.^46^ have shown the selenite absorption in the wheat is suppressed by the metabolic inhibitor CCCP (carbonyl cyanide 3-chlorophenylhydrazone). They stated that phosphate competitively inhibits selenite influx by wheat roots. Se is absorbed by the roots and converted to other forms such as selenomethionine, but are almost insoluble forms. Therefore, Se transformation from the root to the aerial parts in the plants fed by selenite is less than selenate^47^. It is visualized that selenate is activated by the ATP sulfurylase in the form of adenosine 5′-phosphoselenate (APSe)^48^. Selenate entry into the roots is actively against the electrochemical gradient through sulphate transformers in plasma membrane of the root cells. Various studies show that Se absorption and transformation is dependent on the applied Se forms. After Se is absorbed by plants Se is mainly transported into chloroplasts, where the sulfur assimilation is processed^49^. The plants root is able to absorb selenate faster than selenite at a same concentration. Selenate accumulates in plant cells through the active transport. Unlike selenate, there is no evidence that selenite absorption is mediated by membrane carriers^50^. In the plants fed by the selenate, Se is transferred to the aerial parts and selenate is a dominant type in the xylem sap. Conversely, in plants treated with selenite, most Se remains at the roots (a small amount is found in the xylem sap^46^). Se content in maize grain is found to be linearly correlated with Se application rates^39^, that is accordance with our results.

The results of this study showed that sodium selenate and sodium selenite increases the total Phenols and flavonoids contents in *E. amoenum*. Among the experimental treatments, the application of 8 mg L^−1^ sodium selenate significantly increased the total phenols and flavonoids contents. Probably one of the reasons for the increase in the total phenols and flavonoids contents is due to the role of this element in increasing the phenylalanine ammonia lyase (PAL) activity as a key enzyme in the biosynthesis of phenolic and flavonoid compounds in plants^51^. In a research, Se foliar application significantly resulted in increasing the acid ascorbic amount of green tea leaves^19^. Redox properties of phenolic compounds play an important role in the absorption and neutralization of free radicals^52^. However, the mechanism of Se effect on increasing the amount of phenolic and flavonoid compounds in the plants remains unknown. A linear relationship between the total polyphenols content and antioxidant potential has been found in plants of some species before^53^.

In the present study, the use of Se sources improved the antioxidant capacity of *E. amoenum*. When the plants were sprayed with 8 mg L^−1^ sodium selenite and harvested at end of flowering stage, higher antioxidant activity (DPPH radical scavenging) in flowers was observed compared to the other treatments. Poldma et al.^54^ reported a positive correlation between Se foliar application and antioxidant capacity in garlic bulbs. Contrary to the results of the present study, the content of phenolic compounds in the bulbs decreased. In some plants, Se has been reported to cause antioxidant effects and inhibit membrane peroxidation at certain concentrations^17^. Although Se has not yet been found in the structure of the plant glutathione peroxidase (GPX), it is believed that the effectiveness of Se is further enhanced by increased levels of antioxidant enzymes, including (GPX)^55^. Previous studies have reported a positive role for Se in increasing plant antioxidant activity^16,17^. Our results are agreed with Puccinelli et al.^56^ reported that the application of Se significantly affected the antioxidant capacity as well as the total phenol at harvest.

Our finding indicated that 20 composites were discovered containing ɑ-Pinene with sodium selenate. Which are consisting with Meucci et al.^26^ who found A group of volatile organic compounds, positively correlated with consumer liking and flavor intensity, increased following Se treatment of ripe tomato. The impacts of Se on growth features has been previously observed in plant characters such as the photosynthetic pigments, lipid peroxidation, antioxidants capacity, phenols, flavonoids and soluble sugars contents^6^.

Very little is known about the nature of organic forms of Se in soils. The soluble organic Se compounds are liberated through the decay of seleniferous plants. The soil organic matter contained water-soluble and easily recoverable organic Se compounds. The availability of Se in seleniferous soils was found to be correlated with or dependent upon the Se in the organic or humus fraction. Organic forms of Se are probably more soluble under alkaline than under acidic soil conditions^57^. Compared with studies on inorganic Se, there is comparatively much less research to date on the absorption and transport of organic forms of Se by plants. Researcher showed that SeCys and SeMet were both taken up at rates that were some 20-fold higher than those observed for selenate or selenite. Se-amino acids, in particular, are likely to enter plant cells via amino acid transporters (AA Tr.) It has therefore been reasoned that SeCys and SeMet can be taken up by this amino acid transporter as well. As there are many classes of amino acid transporters, it is reasonable to hypothesize that other amino acid transporters will also be involved in the absorption of organic forms of Se, but work in this area remains scant^58^.

Genetic analysis among accessions of Arabidopsis thaliana showed that several genes involved in sulfur (S) assimilation may be responsible for the differences in Se resistance and accumulation, and resistance to selenite and selenate may be regulated by different genes. Molecular and biochemical studies of non-accumulator plants revealed that defense responses mediated by phytohormones (such as ethylene, jasmonic acid, and salicylic acid) play an important role in acquiring Se resistance and accumulation. Production of these phytohormones is enhanced via signal pathways of reactive oxygen species (ROS), and the signal pathways of phytohormones act in a cooperative or antagonistic manner to induce stress and S-uptake and S-metabolic genes^59^.

## Conclusion

Generally, the different stages of flower harvesting had a significant effect on the morpho-physiological and biochemical characteristics of *E. amoenum*. In addition, the application of Se forms and concentrations resulted in changing the quantitative and qualitative characteristics of the plant. In general, the results of this study showed that the use of Se sources to a certain concentration increased the growth characteristics and the use of higher amounts has negative effects on plant growth and development. Foliar application of plants with 4 mg L^−1^ sodium selenate significantly increased plant growth characteristics as well as total alkaloid content. The maximum amount of photosynthetic pigments in the plant was obtained in beginning of flowering stage and after foliar application of 4 mg L^−1^ of sodium selenite. When the plants were sprayed with 8 mg L^−1^ sodium selenite and harvested at end of flowering, higher total phenols and flavonoids contents, antioxidant activity and soluble sugars content was observed. Moreover, foliar application of Se sources significantly increased the content of this element in the petals. Sodium selenate was more effective than sodium selenite in increasing the content of this element.

## Data Availability

The datasets used and/or analyzed during the current study available from the corresponding author on reasonable request.
